# Is Consumption Contagious

**Published:** 1881-02

**Authors:** 


"Is consumption contagious? and can it be transmitted by means of food?" by Herbert C. Clapp, A.M., M.D., Lecturer on Auscultation and Percussion in the Boston Medical School, etc., etc., pp. 178. Boston and Providence. Otis Clapp and Son, 1881.

In these few pages, Dr. Clapp very ably discusses a question of great interest and vital importance. First clearly outlining the scope of his inquiry and defining ambiguous terms, the author proceeds to review this question in all its bearings, and fails not to quote all the testimony, pro and con, available. Thus proving that he seeks only the truth, not to uphold some pet theory. While the entire book should be carefully considered, we would call especial attention to the chapter on *f  d* as a means of infection. If consumption can be thus received, we have here a wide entrance for disease, much more so than sick-room contagion.

As this is such an important question we can not forbear quoting the following, from the introduction: "Some of the more important practical results to be obtained by a judicious agitation of this subject (of contagion, etc.) are these:

(1). That no person, particularly if young, should be allowed to sleep in the same bed, or even (if it can be prevented), in the same room with a consumptive. (2). That no person should be allowed to remain for too long a time in too close, or too constant attendance on a consumptive. (3). That ventilation as perfect as possible be secured. (4). That the most rigid inspection be made of meat that comes into our markets, particularly of the slaughter houses and of all cows that furnish milk."

